# Supplementary material for: Systemic Antibiotics and Obesity: Analyses from a Population-Based Cohort
Source: J Clin Med. 2021 Jun 12;10(12):2601. doi: 10.3390/jcm10122601 (PMC8231544; doi:10.3390/jcm10122601)
Supplement: Supplementary file 1 [file jcm-10-02601-s001.zip › jcm-1232125-supplementary.pdf]

Supplementary Tables

Supplementary Table 1. Antibiotics code classification

Code	classification
610	Systemic antibiotics
611	Mainly acting on gram-positive bacteria
612	Mainly acting on gram-negative bacteria
613	Mainly acting on mycobacterium species
614	Mainly acting on gram-positive bacteria, Rickettsia, and virus
615	Mainly acting on gram-negative bacteria, Rickettsia, and virus
616	Mainly acting on fungus and protozoa
618	Acting on gram-positive and gram-negative bacteria
619	Other antibiotics

Supplementary Table 2. Results of logistic models for risk of obesity in according to the treatment duration of antibiotics against gram-positive and gram-negative bacteria.

Antibiotics against gram-positive bacteria				
	Antibiotics prescription days for 10 years			
	Non-users (N=103,908)	Tertile 1 (N=60,725)	Tertile 2 (N=46,586)	Tertile 3 (N=55,228)
Obesity 1*, OR (95%CI)				
Event	19,663	11,829	9,020	11,368
Model 1**	1.00 (ref.)	1.036 (1.01;1.06)	1.03 (1.00;1.06)	1.11 (1.08;1.14)
Model 2**	1.00 (ref.)	1.07 (1.04;1.09)	1.07 (1.04;1.10)	1.13 (1.11,1.17)
Model 3**	1.00 (ref.)	1.06 (1.03;1.09)	1.06 (1.03,1.09)	1.09 (1.07,1.12
Model 4**	1.00 (ref.)	1.06 (1.03,1.09)	1.06 (1.03,1.09)	1.09 (1.06,1.13)
Obesity 2*, OR (95%CI)				
Event	39,902	23,792	17,872	22,043
Model 1**	1.00 (ref.)	1.03 (1.01;1.06)	0.99 (0.98,1.02)	1.07 (1.04,1.09)
Model 2**	1.00 (ref.)	1.07 (1.05;1.09)	1.05 (1.03;1.08)	1.11 (1.09,1.14)
Model 3**	1.00 (ref.)	1.07 (1.04;1.09)	1.04 (1.01;1.06)	1.07 (1.05,1.09)
Model 4**	1.00 (ref.)	1.07 (1.04;1.09)	1.04 (1.01,1.06)	1.07 (1.05,1.09)
Obesity 3*, OR (95%CI)				
Event	36,902	21,879	16,298	19,811
Model 1**	1.00 (ref.)	1.02 (1.00,1.04)	0.98 (0.96;1.00)	1.02 (0.99;1.04)

Model 2**	1.00 (ref.)	1.07 (1.04,1.09)	1.05 (1.02;1.07)	1.09 (1.07;1.12)
Model 3**	1.00 (ref.)	1.06 (1.04;1.08)	1.03 (1.01;1.05)	1.06 (1.03;1.08)
Model 4**	1.00 (ref.)	1.06 (1.04;1.08)	1.03 (1.01;1.05)	1.06 (1.03;1.08)
Obesity 4*, OR (95%CI)				
Event	22,663	13,742	10,594	13,600
Model 1**	1.00 (ref.)	1.05 (1.02;1.07)	1.06 (1.03;1.08)	1.17 (1.14;1.20)
Model 2**	1.00 (ref.)	1.07 (1.05,1.09)	1.08 (1.05;1.11)	1.15 (1.12,1.17)
Model 3**	1.00 (ref.)	1.06 (1.04;1.09)	1.06 (1.03;1.09)	1.10 (1.08;1.13)
Model 4**	1.00 (ref.)	1.06 (1.04,1.09)	1.06 (1.04,1.09)	1.11 (1.08,1.13)

Antibiotics against gram-nagetive bacteria				
	Antibiotics prescription days for 10 years			
Obesity 1*, OR (95%CI)	Non-users (N=6,933)	Tertile 1 (N=85,813)	Tertile 2 (N=86,537)	Tertile 3 (N=87,164)
Event	1,299	16,025	16,515	18,041
Model 1**	1.00 (ref.)	0.99 (0.94;1.06)	1.02 (0.96;1.09)	1.13 (1.06;1.21)
Model 2**	1.00 (ref.)	1.07 (1.01,1.14)	1.15 (1.08;1.23)	1.29 (1.21;1.38)
Model 3**	1.00 (ref.)	1.07 (0.99;1.14)	1.13 (1.06,1.21)	1.22 (1.15,1.31)
Model 4**	1.00 (ref.)	1.07 (0.99;1.14)	1.13 (1.06;1.21)	1.23 (1.15;1.31)
Obesity 2*, OR (95%CI)				
Event	2,658	32,757	33,156	35,038

Model 1**	1.00 (ref.)	0.99 (0.94;1.04)	0.99 (0.95;1.05)	1.08 (1.03;1.14)
Model 2**	1.00 (ref.)	1.09 (1.03;1.14)	1.16 (1.10;1.22)	1.29 (1.22;1.36
Model 3**	1.00 (ref.)	1.08 (1.03;1.14)	1.14 (1.08;1.20)	1.23 (1.17;1.29)
Model 4**	1.00 (ref.)	1.08 (1.03;1.14)	1.14 (1.08;1.20)	1.23 (1.17,1.29)
Obesity 3*, OR (95%CI)				
Event	2,491	30,402	30,443	31,554
Model 1**	1.00 (ref.)	0.98 (0.93;1.03)	0.97 (0.92;1.02)	1.01 (0.96;1.07)
Model 2**	1.00 (ref.)	1.07 (1.01;1.13)	1.14 (1.08;1.20)	1.25 (1.19,1.31)
Model 3**	1.00 (ref.)	1.06 (1.01,1.12)	1.12 (1.06;1.18)	1.19 (1.13,1.26)
Model 4**	1.00 (ref.)	1.06 (1.01,1.12)	1.12 (1.06;1.18)	1.19 (1.13,1.25)
Obesity 4*, OR (95%CI)				
Event	1,466	18,380	19,228	21,525
Model 1**	1.00 (ref.)	1.02 (0.96;1.08)	1.07 (1.00,1.13)	1.22 (1.15,1.29
Model 2**	1.00 (ref.)	1.09 (1.03,1.16)	1.17 (1.10,1.25)	1.33 (1.25;1.41)
Model 3**	1.00 (ref.)	1.08 (1.02,1.15)	1.15 (1.08;1.23)	1.26 (1.18;1.34)
Model 4**	1.00 (ref.)	1.09 (1.02,1.15)	1.16 (1.09,1.23)	1.27 (1.19,1.35)

*Obesity 1: subjects who satisfied both BMI and WC criteria; *Obesity 2: subjects who satisfied BMI or WC criteria; *Obesity 3: subjects who satisfied only BMI criteria; *Obesity 4: subjects who satisfied only WC criteria.

**Model 1: non-adjusted; **Model 2: adjusted for age and sex; **Model 3: adjusted for factors in Model 2 and comorbidity such as DM, HTN, and dyslipidemia; **Model 4: adjusted for factors in Model 3 and lifestyle factors such as drinking, smoking, and exercise.
